# CTSG-expressing mast cells confer resistance to immunotherapy in colorectal cancer

**DOI:** 10.3389/fonc.2026.1854253

**Published:** 2026-07-01

**Authors:** Xuehui Jiang, Runsheng Hong, Zilin Liu, Chao Zhong, Yun Dai

**Affiliations:** 1 Department of Gastroenterology, Peking University First Hospital, Beijing, China; 2 Department of Ultrasound, Peking University First Hospital, Beijing, China; 3 Department of Immunology, School of Basic Medical Sciences, NHC Key Laboratory of Medical Immunology, Medicine Innovation Center for Fundamental Researches on Major Immunology-Related Diseases, Peking University, Beijing, China

**Keywords:** cathepsin G, colorectal cancer, immunotherapy, mast cell, tumor microenvironment

## Abstract

**Background:**

Mast cells are increasingly recognized as an important regulator of the tumor microenvironment. However, their role in colorectal cancer (CRC) is controversial, and there is a lack of targetable mast cell-specific molecules to improve the tumor microenvironment and overcome resistance to immunotherapy.

**Methods:**

We used single-cell RNA sequencing, bulk RNA sequencing, and bioinformatics analyses to characterize mast cell subsets in the CRC tumor microenvironment and assess their association with immunotherapy response. We further employed a murine orthotopic CRC model and multi-omics approaches to explore how mast cells regulate anti-tumor immunity.

**Results:**

Tumor-infiltrating mast cells displayed altered transcriptional state in CRC and closely associated with tumor molecular features. Mast cell deficiency significantly attenuated tumor growth and increased immune cell infiltration in an orthotopic CRC model. Strikingly, a subset of mast cells specifically expressing cathepsin G (CTSG) correlated with immunotherapy resistance and poor prognosis in patient with CRC. Mast cell-derived CTSG contributed to tumor metabolic adaptation and limited chemokine-mediated immune recruitment. The interaction between mast cells and tumor cells was mainly mediated via protease-activated receptor 2 (PAR2) signaling. Furthermore, pharmacological inhibition of CTSG improved the recruitment and function of CD8^+^ T cell and enhanced the efficacy of anti-PD-1 *in vivo*.

**Conclusion:**

Our findings uncover a pro-tumoral CTSG^+^ mast cell subset associated with a metabolically active and immune-restricted microenvironment in CRC, and highlight CTSG as a promising target for improving immunotherapy response.

## Introduction

Colorectal cancer (CRC) is the third most common cancer and the second leading cause of cancer-related death worldwide (1). Immune checkpoint inhibitors (ICIs) have achieved substantial clinical success in multiple solid tumors, but their efficacy in CRC remains very limited (2). Clinical responses to ICIs are restricted to CRC characterized by high microsatellite instability or deficient DNA mismatch repair (MSI-H/dMMR) (3, 4), which account for only 10%-15% cases. However, the majority of CRC (85%-90%) with microsatellite stability or proficient mismatch repair (MSS/pMMR) are largely resistance to ICI therapy (5). The immunosuppressive tumor microenvironment (TME) is a key obstacle affecting ICI response in CRC with MSS/pMMR (6).

Mast cells are long-lived tissue-resident innate immune cells, infiltrating in multiple solid tumors particularly CRC, breast cancer, and melanoma (7). Mast cells are traditionally well-known for their roles in protection against parasitic infections and in allergic responses (8). Within the TME, mast cells extensively interact with tumor cells, immune cells, and extracellular matrix through direct cell-to-cell interactions or release of diverse mediators, including proteases, histamine, lysosomal hydrolases and cytokines. Recent single-cell transcriptomic studies have revealed mast cell heterogeneity and plasticity in solid tumors, and their roles are highly dependent on distinct functional subsets and the characteristic molecular features (9, 10). Targeting mast cells could be a promising approach to improve the TME and overcome resistance to immunotherapy.

The gastrointestinal tract comprises the largest population of mast cells in the body. However, the role of mast cells in CRC is controversial as they can either promote or inhibit it (11). Several studies have shown that high mast cell density in CRC was correlated with favorable prognosis (12), whereas others documented mast cells enriched in CRC directly interacted with tumor cells to promote invasion and progression (13). These seemingly conflicting findings highlight the need to recognize the functional heterogeneity of mast cell in CRC. Moreover, targeting mast cells to enhance ICI efficacy remains challenging due to a lack of specific molecules responsible for the immunosuppressive microenvironment.

Cathepsin G (CTSG) is a member of the cathepsin family, which was initially found to be secreted by activated neutrophils and acts as a serine protease to clear pathogens. CTSG is also expressed in mast cells and monocytes, regulating cytokine activation and antigen presentation (14, 15). However, current evidence for its immune regulatory roles is largely derived from *in vitro* studies, and the involvement of mast cell-derived CTSG in regulating the TME remains unclear.

In the present study, we identified a subset of CTSG^+^ mast cells associated with poor ICI response in CRC. Mast cell-derived CTSG promoted tumor metabolic adaptation and limited CD8^+^ T cell recruitment in the TME. Furthermore, pharmacological inhibition of CTSG can enhance the efficacy of anti-PD-1 and improve the recruitment and function of CD8^+^ T cell in orthotopic CRC model. Our findings uncover a previously underappreciated CTSG^+^ mast cell-related program in CRC and highlight CTSG as a potential therapeutic target to overcome immunotherapy resistance.

## Materials and methods

### Cell lines and *in vitro* treatment

The murine colon adenocarcinoma cell lines MC38 and luciferase-labeled MC38 (MC38-Luc) were obtained from the National Infrastructure of Cell Line Resource (China). Cells were cultured in RPMI-1640 medium supplemented with 10% fetal bovine serum (FBS) and 1% penicillin-streptomycin in a humidified incubator at 37 °C with 5% CO_2_. Cells were routinely tested and confirmed to be free of mycoplasma contamination. All experiments were performed using cells within 3–5 passages after thawing. For *in vitro* experiments, MC38 cells were cultured in serum-free RPMI-1640 medium and treated with purified cathepsin G (0.02 U/mL) or solvent control for 16 h.

### Mice and tumor models

All animal experiments were approved by the Institutional Animal Care and Use Committee of Peking University First Hospital. *Kit^W-sh/W-sh^* mice were maintained on a C57BL/6 background, and age- and sex-matched C57BL/6 wild-type mice were used as controls. Both strains were purchased from The Jackson Laboratory (stock numbers 012861 and 000664, respectively). Mice were housed under specific pathogen-free conditions with a 12h light/dark cycle.

For the orthotopic CRC model, mice were anesthetized with inhalational isoflurane, induced with 4%-5% isoflurane in an induction chamber and maintained at 1%-2% via a nose cone with a continuous oxygen flow of 1 L/min. Anesthetic depth was monitored throughout the procedure by assessing respiratory rate and pedal withdrawal reflex, defined as the absence of limb withdrawal in response to a gentle toe pinch. Following a midline laparotomy, the cecum was exteriorized, and 50 μL of the MC38-Luc cell suspension (2 × 10^7^ cells/mL) was slowly injected into the mesenteric side of the cecum using a fine-gauge needle. Tumor growth was monitored weekly using bioluminescence imaging. Mice were anesthetized with isoflurane and intraperitoneally injected with D-luciferin potassium salt (150 mg/kg, Beyotime, ST196). Imaging was performed using an IVIS Spectrum system (PerkinElmer). All images within the same experiment were displayed using an identical bioluminescence intensity scale. Total photon flux was calculated within a defined region of interest (ROI) using Living Image software as a semi-quantitative surrogate measure of tumor burden, and the same ROI placement strategy and analysis parameters were applied to all mice.

For immune checkpoint blockade and CTSG inhibitor therapy, MC38-Luc tumor-bearing mice were intraperitoneally injected with 100 μg of anti-PD-1 (Bioxcell, BE0273) or isotype antibody (Bioxcell, BE0089) on day 10 and day 14 post tumor implantation. Cathepsin G Inhibitor I (MedChemExpress, HY-103351) was intraperitoneally injected at 10 mg/kg starting from day 3 post tumor inoculation, and injections were repeated every 2 days.

The experimental endpoint was defined as the pre-specified time point for tissue collection. Humane endpoints were independently applied, including >15%-20% body weight loss, marked abdominal distension, reduced mobility, persistent anorexia, signs of intestinal obstruction, or severe distress. At the experimental endpoint or when humane endpoint criteria were met, mice were euthanized by CO_2_ inhalation followed by cervical dislocation, and tumor tissues were harvested for subsequent analyses.

### Immune cell isolation and flow cytometry analysis

Tumor tissues were minced and digested with collagenase IV (0.5 mg/mL), DNase I (0.5 mg/mL), and dispase II (3 mg/mL) at 37 °C for 20 min. The resulting cell suspension was filtered through a 70-μm strainer and washed with PBS. Immune cells were enriched by Percoll density gradient centrifugation. For flow cytometry analysis, cells were stained with eBioscience™ Fixable Viability Dye eFluor™ 506 to exclude dead cells, and then incubated with anti-CD16/32 antibody for Fc receptor blocking for 10 min at 4 °C. Surface staining was performed in FACS buffer (PBS with 2% FBS) for 30 min at 4 °C. For intracellular cytokine staining, cells were stimulated with PMA (50 ng/mL)/ionomycin (1 μg/mL) in the presence of brefeldin A (5 μg/mL) for 4 h, followed by fixation and permeabilization using an Intracellular Fixation & Permeabilization kit (eBioscience).

### Public database analysis

Transcriptomic and clinical data for CRC patients were obtained from The Cancer Genome Atlas (TCGA). Immune cell infiltration was estimated using CIBERSORT, and immunotherapy response was predicted using the Tumor Immune Dysfunction and Exclusion (TIDE) algorithm. Survival analysis was performed using the Kaplan-Meier Plotter database, which integrates gene expression and clinical data from 17 independent CRC cohorts and includes 2,137 tumor samples (16). Patients were stratified into CTSG-high and CTSG-low groups according to the median CTSG expression level across tumor samples.

### Single-cell RNA-seq data processing and analysis

Publicly available Single-cell Colorectal Cancer Atlas (https://crc.icbi.at/) (17) was used to compare immune cell features between adjacent normal and primary tumor tissues and to analyze mast cell proportions in CRC tumors by MMR/MSI/CMS/immunotherapy response (RECIST). Publicly available scRNA-seq datasets GSE236581 of CRC patients treated with ICIs were obtained from previously published studies (18). These datasets had been preprocessed, integrated, and annotated by the original authors, and the accompanying metadata were used for downstream analyses. Mast cells were extracted based on the original cell-type annotations provided in the metadata. Downstream analyses were performed using Seurat (v5). Cells were filtered based on quality control metrics (500–5000 UMIs and <10% mitochondrial genes). Data were normalized and scaled, and the top 2,000 variable genes were used for principal component analysis. Mast cells were clustered using the first 25 principal components (**Supplementary Figure 1A**). A range of clustering resolutions from 0.1 to 1.0 was evaluated, and a resolution of 0.4 was selected (**Supplementary Figures 1B, C**). Mast cell subpopulations were annotated based on differentially expressed genes, canonical mast cell markers, and functional marker programs. Potential contamination by epithelial, stromal, and other immune cells was assessed using representative lineage markers (**Supplementary Figure 1D**). Patients with CRC were stratified into MC_CTSG_high and MC_CTSG_low groups according to the median proportion of MC_CTSG subset among total mast cells. Pseudo-bulk differential expression analysis was performed by aggregating gene counts at the sample level, followed by differential analysis using the limma package with batch effects included as covariates. Cell-cell communication analysis was conducted using CellChat. Gene set enrichment analysis (GSEA) was performed using the R package clusterProfiler. Leading-edge genes were extracted from the selected GSEA pathways, and up to five leading-edge genes with significant differential expression and high ranks were retained. Expression values were standardized to row-wise z-scores across patients and visualized as heatmaps.

### Bulk RNA-seq

For tumor tissue bulk RNA-seq, MC38-Luc tumor-bearing mice were euthanized, and tumor tissues were excised, snap-frozen in liquid nitrogen, and stored at -80 °C until further processing. For *in vitro* study, MC38 cells were harvested at the indicated time points after CTSG treatment, washed twice with cold PBS and lysed in TRIzol reagent. Total RNA was extracted using TRIzol reagent followed by column-based purification with the SteadyPure RNA Extraction Kit (Accurate Biotechnology, AG21024). Libraries were prepared using the TruSeq Stranded mRNA Library Prep Kit (Illumina) and sequenced on the Illumina NovaSeq 6000 platform (150 bp paired-end reads). Raw reads were processed using fastp, aligned to the mouse reference genome (mm10) using HISAT2, and gene counts were generated using HTSeq-count. Differential gene expression analysis was performed using DESeq2. GSEA was performed using the clusterProfiler. CD8^+^ T cell Functional states were evaluated using ssGSEA-based signature scoring, including activation, cytotoxicity, IFN-γ response, cytokine secretion, chemotaxis, and exhaustion signatures (**Supplementary Table 1**). Immune cell infiltration was estimated using Immune Cell Abundance Identifier for mouse (ImmuCellAI-mouse) as previously reported (19).

### Untargeted proteomics analysis

Protein samples were analyzed by liquid chromatography-tandem mass spectrometry (LC-MS/MS) using a timsTOF Pro 2 system (Bruker). Raw data were processed using MaxQuant software for protein identification and quantification. Differentially expressed proteins were defined as those with |log_2_FC| > 0.25 and p < 0.05. In addition, partial least squares discriminant analysis (PLS-DA) was performed, and proteins with a variable importance in projection (VIP) score > 1 were considered key contributors. GSEA was performed using the clusterProfiler.

### Statistical analysis

GraphPad Prism5 software (GraphPad Software, Inc, San Diego, CA, USA) and R software (4.5.2) were used for graphic representation and statistical analysis. Difference was analyzed by parametric (two-tailed Student’s *t*-test) or nonparametric (Mann-Whitney *U* test) test. One-way ANOVA and Dunnett *t*-test were used for multiple comparisons. Survival curves were compared using a log-rank Mantel-Cox test. Data are presented as mean ± SD for normally distributed data or as median with interquartile range for non-normally distributed data. A *p* value of less than 0.05 was considered statistically significant.

## Results

### 1 Mast cells affect the immune microenvironment and tumor progression in CRC

**Figure 1:**
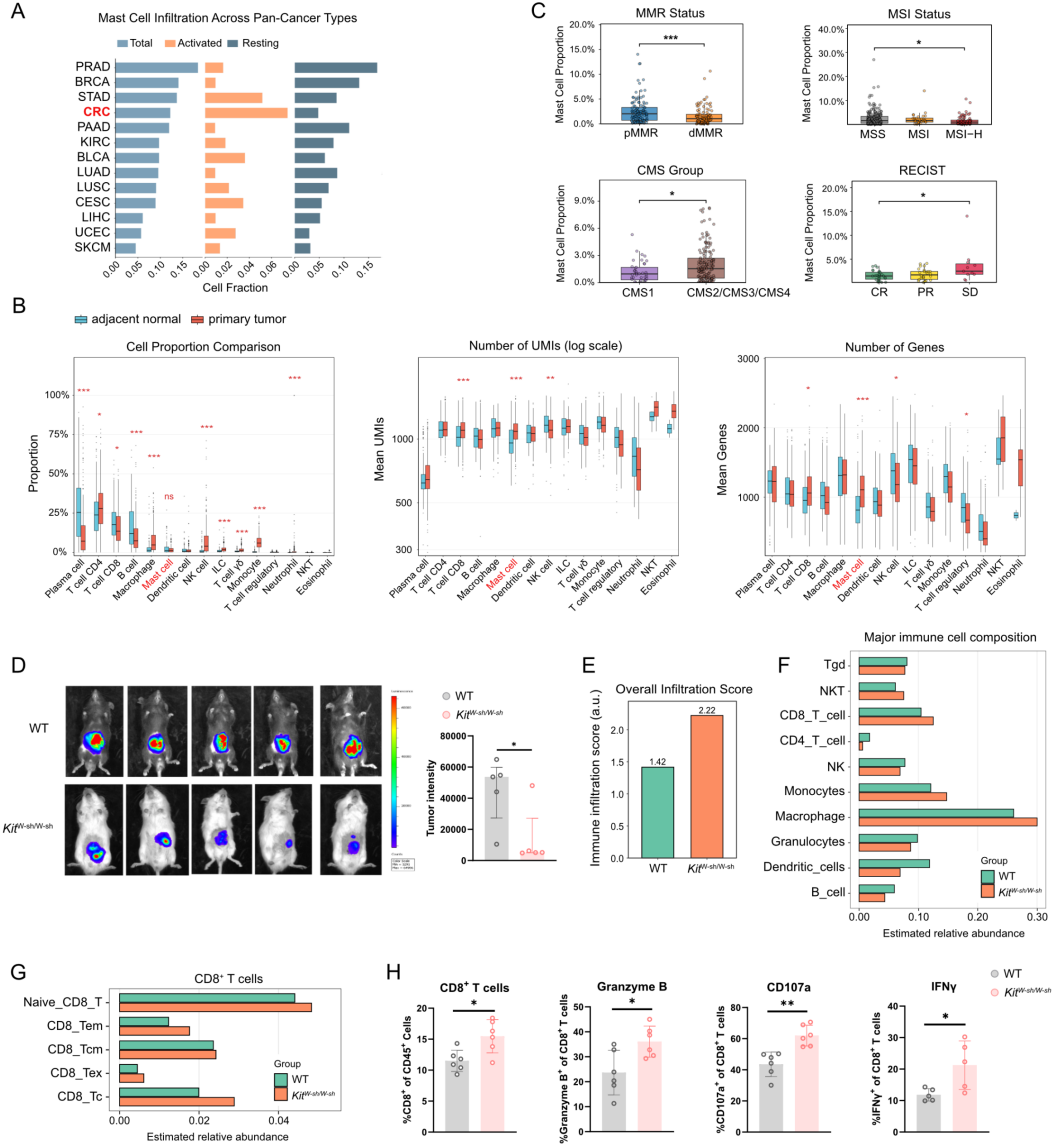
Mast cells affect the immune microenvironment and tumor progression in CRC **(A)** Comparison of the estimated fractions of total, activated, and resting mast cells across multiple tumor types in The Cancer Genome Atlas (TCGA) dataset using the CIBERSORT immune-cell categorization. **(B)** single-cell RNA sequencing (scRNA-seq) datasets (17) were used to compare immune cell proportions and transcriptional features between tumors and adjacent normal tissues. Left, proportions of the indicated immune cell populations. Middle, numbers of unique molecular identifiers (UMIs) per cell, shown on a log scale. Right, numbers of detected genes per cell. **(C)** Box plots showing mast cell proportions in CRC tumor tissues from scRNA-seq datasets (17) according to MMR status, MSI status, CMS group, and immunotherapy response (RECIST). CR, complete response; PR, partial response; SD, stable disease. Each dot indicates one sample. **(D)** Orthotopic colorectal tumor model was established by inoculating MC38-Luc cells into the mesenteric side of the cecum of *Kit^W-sh/W-sh^* and wild-type (WT) C57BL/6 mice. Tumor growth was monitored by *in vivo* bioluminescence imaging. Representative images show tumor size and total photon flux within the tumor region. Bars indicate the median with interquartile range. Statistical significance was assessed using the two-sided Mann–Whitney U test. *P < 0.05. **(E–G)** Immune cell infiltration was estimated using ImmuCellAI-mouse based on bulk RNA-seq data. Bars represent the estimated infiltration scores for each immune cell type. **(E)** Overall immune infiltration scores in tumors from WT and *Kit^W-sh/W-sh^* mice, calculated as the sum of the estimated relative enrichment scores of major immune cell types. a.u., arbitrary units. **(F)** Estimated relative infiltration scores of major immune cell populations in the two groups. **(G)** Estimated relative infiltration scores of CD8^+^ T cell subsets. **(H)** Flow cytometric analysis of CD8^+^ T cell frequency among CD45^+^ cells and the proportions of Granzyme B^+^, CD107a^+^, and IFNγ^+^ cells within CD8^+^ T cells in tumors from WT and *Kit^W-sh/W-sh^* mice. Each dot represents one mouse. Bars indicate the mean ± SD. Statistical significance was assessed using the two-sided unpaired *t* test. *P < 0.05, **P < 0.01.

We first used CIBERSORT to analyze mast cell infiltration patterns across cancer types in TCGA and observed that CRC showed a relatively high abundance of activated mast cells (**Figure 1A**). Analyses of an integrated scRNA-seq dataset of CRC (17) showed that although the proportion of mast cells did not significantly differ between adjacent normal and primary tumors tissues, both the unique molecular identifier (UMI) counts and the number of genes were markedly increased in tumor-infiltrating mast cells (**Figure 1B**), indicating increased transcriptomic complexity of mast cells within the TME. Specifically, mast cells were significantly enriched in pMMR/MSS tumors (**Figure 1C**). Given that CMS1 tumors are typically MSI-enriched and immune-infiltrated, whereas CMS2, CMS3, and CMS4 generally lack this MSI-like immune-reactive phenotype, we further examined mast cell infiltration across CMS groups. Mast cell infiltration was higher in CMS2, CMS3, and CMS4 tumors than in CMS1 tumors (**Figure 1C**). Among CRC patients receiving ICI therapy, mast cell infiltration in tumors from patients with complete response was significantly lower than that from patients with stable disease (**Figure 1C**). These findings suggest that mast cell abundance closely associates with the tumor molecular features and influences the efficacy of ICI in CRC.

To investigate the role of mast cells within the TME, we established an orthotopic colon tumor model and found that the tumor burden was significantly reduced in mast cell-deficient (*Kit^W-sh/W-sh^*) mice compared to wild-type (WT) mice (**Figure 1D**), indicating a tumor-promoting role of mast cells. To explore the potential mechanisms of mast cell-mediated regulation of the TME, we performed bulk RNA-seq on tumors from WT and mast cell-deficient mice. By using ImmuCellAI-mouse tool, we estimated the relative abundance of tumor-infiltrating immune cell populations from bulk gene expression data (19). As shown in **Figure 1E**, tumors from *Kit^W-sh/W-sh^* mice showed a trend toward higher immune infiltration score. Mast cell deficiency was associated with increased estimated abundance of tumor-infiltrating CD8^+^ T cells, NKT cells and monocyte/macrophages, and decreased in CD4^+^ T cells, dendritic cells and B cells (**Figure 1F**). Given the known importance of macrophages in the TME, we further examined the M1- and M2-like macrophage and found that although total macrophage abundance was higher in tumors from *Kit^W-sh/W-sh^* mice, no clear shift in macrophage polarization was observed (**Supplementary Figure 2A**). Further subset analysis of CD8^+^ T cells revealed an increase in effector memory (CD8_Tem) and cytotoxic T cell (CD8_Tc) populations (**Figure 1G**). Consistently, flow cytometric analysis confirmed increases in tumor-infiltrating CD8^+^ T cell frequency and their Granzyme B, CD107a, and IFNγ expression in *Kit^W-sh/W-sh^* mice when compared with WT mice (**Figure 1H**). Gene set enrichment analysis (GSEA) demonstrated that pathways upregulated in tumors from *Kit^W-sh/W-sh^* mice were predominantly associated with immune responses, including innate immune response, leukocyte chemotaxis and T cell activation (**Supplementary Figure 2B**). Kyoto Encyclopedia of Genes and Genomes (KEGG) analysis similarly identified enrichment of T cell receptor signaling, cytokine-cytokine receptor interaction and chemokine-mediated pathways in tumors from *Kit^W-sh/W-sh^* mice (**Supplementary Figure 2C**). Visualization of the core-enrichment genes further showed that the leading genes contributing to these enriched pathways were consistently upregulated in *Kit^W-sh/W-sh^* tumors (**Supplementary Figure 2D****).** In contrast, pathways downregulated in *Kit^W-sh/W-sh^* tumors were predominantly related to metabolic processes and mitochondrial biosynthesis, including oxidative phosphorylation, glycolysis/gluconeogenesis, tricarboxylic acid cycle and glutathione metabolism, with coordinated changes in the top core-enrichment genes, including *Idh2, Ndufa2 and Sod1* (**Supplementary Figure 2D**). These findings suggest that mast cells could facilitate tumor progression via regulating the TME.

### 2 CTSG^+^ mast cells are associated with immunotherapy resistance in CRC

**Figure 2:**
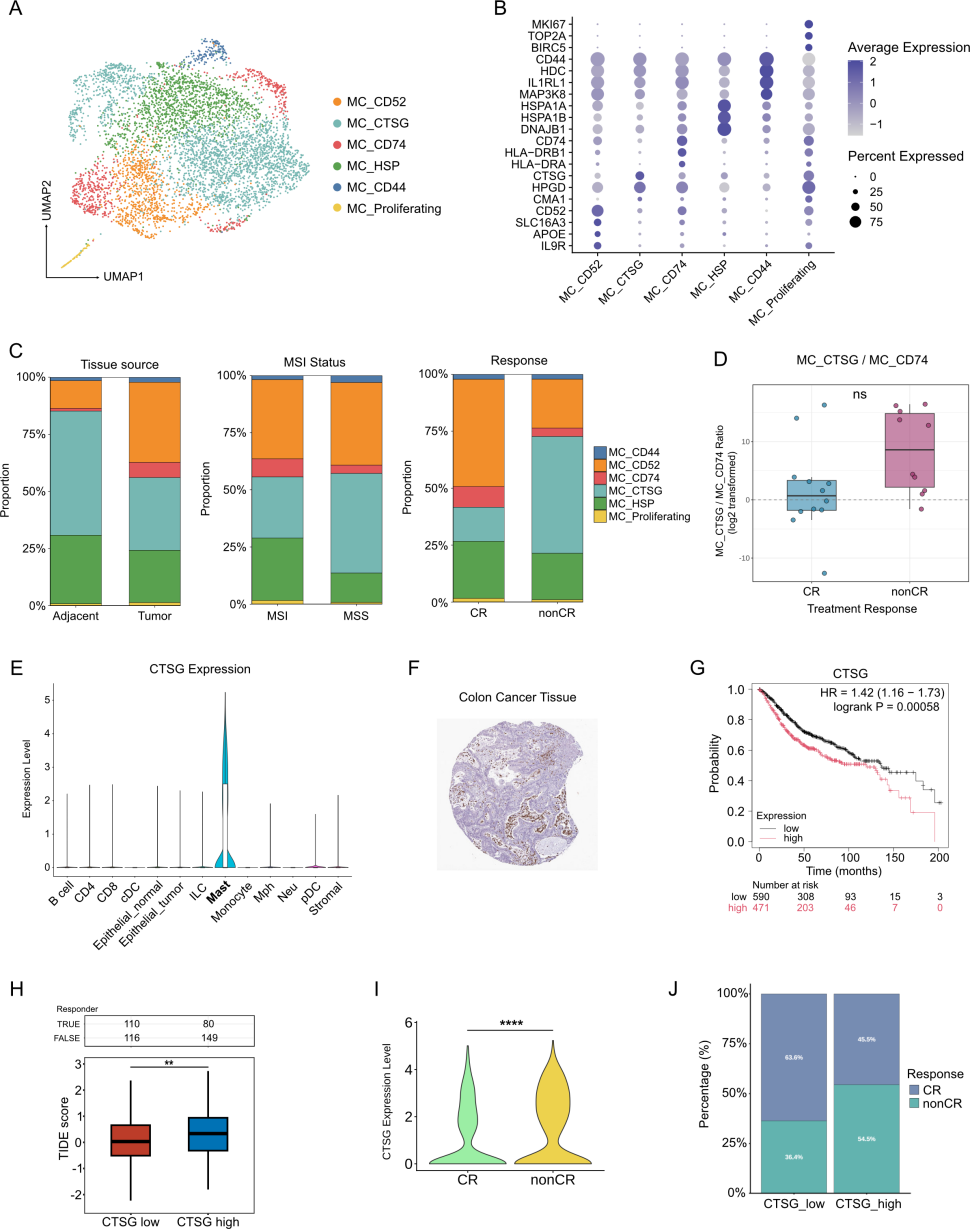
CTSG^+^ mast cells are associated with immunotherapy resistance in CRC **(A)** UMAP visualization of mast cell subsets in CRC based on scRNA-seq data (18). **(B)** Dot plot showing marker genes across clusters from **(A)**. **(C)** Mast cell subset distribution in tumor vs. matched adjacent normal tissues (left), MSS vs. MSI-H tumors (middle), and patients with complete response (CR) vs. non-complete response (non-CR) to ICI therapy (right). **(D)** Ratio of MC_CTSG to MC_CD74 subsets in tumors from patients with CR and non-CR to ICI therapy. Each dot represents one patient. ns, not significant. **(E)** Expression of CTSG across different cell types in CRC. **(F)** Immunohistochemical staining of CTSG in CRC tissues from the Human Protein Atlas (HPA) database. **(G)** Kaplan-Meier overall survival (OS) curves of patients with CRC stratified by median CTSG expression (high vs. low) based on data from the Kaplan-Meier Plotter database (16). **(H)** Tumor Immune Dysfunction and Exclusion (TIDE) scores in tumors stratified by median CTSG expression. **(I)** Pseudo-bulk analysis of CTSG expression in tumors from patients with CR and non-CR following ICI treatment based on scRNA-seq data (18). **(J)** Distribution of immunotherapy responses in patients stratified by median CTSG expression level (high vs. low) derived from pseudo-bulk analysis of scRNA-seq data.

Recent studies have revealed the heterogeneity of tumor-infiltrating mast cells (10, 20), we therefore analyzed mast cell subsets in CRC to explore their association with tumor molecular features and immunotherapy response using a published scRNA-seq dataset (18). By unsupervised clustering, we identified six transcriptionally distinct mast cell subsets based on their canonical markers and differential gene expression. (**Figures 2A, B**; **Supplementary Figures 3A, B**). The MC_CD52 subset was characterized by high expression of *CD52* and *IL9R*, along with key genes involved in glucose and lipid metabolism, suggesting a metabolism-associated transcriptional feature. The MC_CTSG subset expressed high levels of protease-related genes including *CTSG* and *CMA1*, consisting with a protease-enriched connective tissue-like phenotype. The MC_CD74 subset was enriched for MHC class II molecules and *CD74*, consisting with antigen-presentation features. In addition, MC_HSP showed high expression of heat shock protein genes, MC_CD44 showed high expression of *CD44* and *HDC*, and MC_Proliferating corresponded to actively cycling mast cells. In patients with CRC, the distribution of these subsets markedly differed between tumors and matched adjacent normal tissues (**Figure 2C**; **Supplementary Figure 4A****).** Notably, the MC_CTSG subset was significantly enriched in MSS tumors compared to MSI tumors (**Figure 2C**; **Supplementary Figure 4A**). In CRC patients receiving ICIs, the proportion of CTSG^+^ mast cells was higher in non-complete response (non-CR) group than in complete response (CR) group (**Figure 2C**; **Supplementary Figure 4A**). CD74^+^ mast cells were reported to be associated with favorable response to immunotherapy in hepatocellular carcinoma, melanoma and triple-negative breast cancer (20, 21). We found that MC_CD74 subset was slightly decreased, while the ratio of MC_CTSG to MC_CD74 showed a higher trend in the non-CR group (**Figure 2D**), indicating that the heterogeneity of mast cell is associated with ICI response patterns in CRC.

In CRC tumor tissues, CTSG expression was specifically enriched in mast cells (**Figure 2E**). Immunohistochemical analysis from the Human Protein Atlas also confirmed that CTSG predominantly localized within the tumor stroma (**Figure 2F**). Survival analysis using the Kaplan-Meier Plotter database (16) revealed that high CTSG expression was significantly associated with poor prognosis in patients with CRC (**Figure 2G**). Moreover, tumors with high CTSG level exhibited higher TIDE scores, suggesting an increased potential for immune evasion (**Figure 2H**). We further analyzed single-cell transcriptomics data (18) to determine the impact of mast cell-derived CTSG on ICI efficacy in CRC. The expression level of CTSG in mast cells was significantly higher in non-CR than in CR patients (**Figure 2I**), and the proportion of non-CR patients with high CTSG expression was significantly increased (**Figure 2J**), supporting a potential link between CTSG-enriched mast cells and reduced ICI responsiveness.

### 3 Mast cells-derived CTSG modulates the tumor microenvironment via PAR signaling

**Figure 3:**
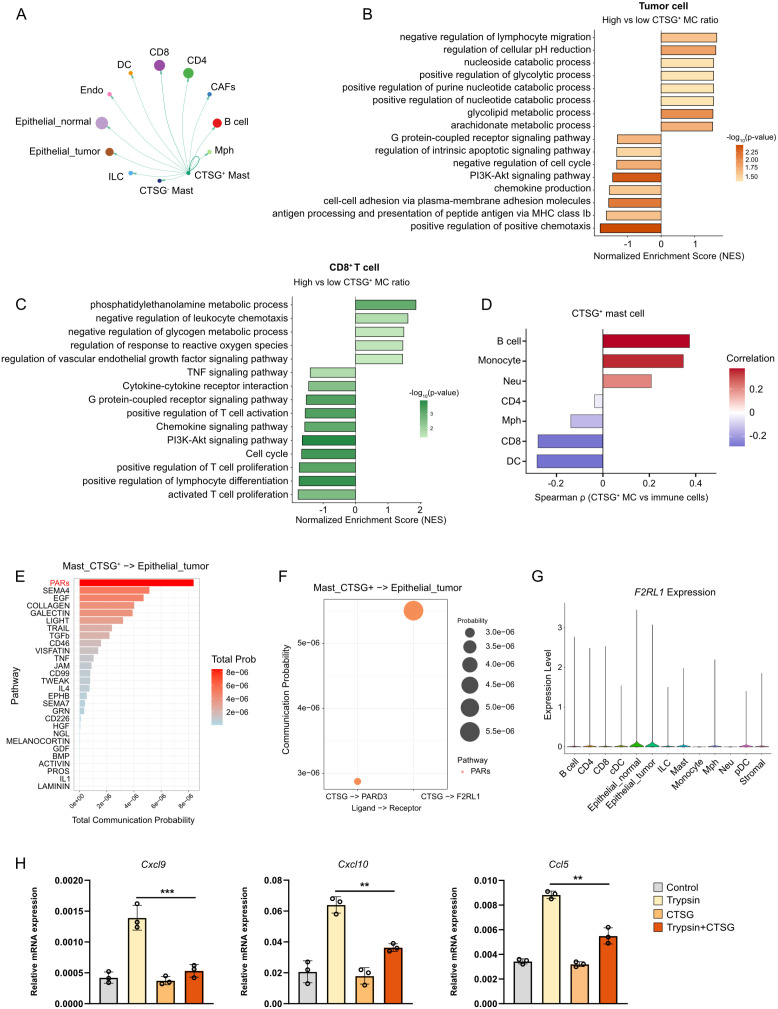
Mast cells-derived CTSG modulates the tumor microenvironment via PAR signaling **(A)** Predicted cell-cell interaction network of CTSG^+^ mast cells with other cell types inferred by CellChat. **(B, C)** GO-based GSEA of tumor cells **(B)** and CD8^+^ T cells **(C)** between MC_CTSG_high and MC_CTSG_low groups, which stratified by median proportion of MC_CTSG cells among total mast cells. Bars represent normalized enrichment scores (NES), and color indicates statistical significance as shown in the plot. **(D)** Bar plot showing Spearman correlation coefficients (ρ) between the abundance of CTSG^+^ mast cells and indicated immune cell populations across samples. **(E)** Inferred signaling pathways between CTSG^+^ mast cells and tumor cells. **(F)** Interaction strength of PAR family signaling pathways between CTSG^+^ mast cells and tumor cells. **(G)** Expression of CTSG across different cell types in CRC. **(H)** Relative mRNA expression of *Cxcl9, Cxcl10* and *Ccl5* in tumor cells treatment with CTSG and/or trypsin. Data shown as mean ± SD and is representative of three independent experiments. Statistical significance was determined by two-sided unpaired *t* test. **P < 0.01, ***P < 0.001.

To explore CTSG-mediated intercellular communication, we performed CellChat analysis using CTSG^+^ mast cells as signal senders and observed widespread interactions in the TME. These interactions were prominently directed toward tumor epithelial cells and key immune populations, including CD8^+^ T cells and macrophages (**Figure 3A**). Tumor cells constitute the dominant cellular component of the TME, while CD8^+^ T cells serve as the primary effectors of antitumor immunity. We therefore investigated the impact of CTSG^+^ mast cells on tumor cells and CD8^+^ T cells by using single-cell transcriptomics datasets (18). Patients with CRC were classified into MC_CTSG_high and MC_CTSG_low groups according to the median proportion of MC_CTSG subset among total mast cells, and GSEA was performed on tumor cells and CD8^+^ T cells between the two groups. In tumor cells, Gene Ontology (GO) analysis revealed that pathways upregulated in the MC_CTSG_high group were predominantly enriched in metabolic processes, including nucleoside and nucleotide catabolism, glycolytic regulation and lipid metabolism (**Figure 3B**), with coordinated changes in the top core-enrichment genes including *SLC4A4, HIF1A* and *ZBTB20* (**Supplementary Figure 5A**). In contrast, pathways associated with chemokine production, positive chemotaxis, antigen processing and presentation, apoptotic signaling and cell-cell adhesion were suppressed in MC_CTSG_high group, accompanied by reduced expression of core-enrichment genes included *CXCL8, ITGA2, HLA-F* and *MDM2* (**Supplementary Figure 5A**). In CD8^+^ T cells, GO enrichment analysis demonstrated a distinct pattern, with pathways related to immune response, such as T cell activation, proliferation, differentiation, and chemokine signaling were reduced in MC_CTSG_high group (**Figure 3C**). Key immune signaling pathways, including cytokine-cytokine receptor interaction and TNF signaling, were also downregulated. Representative core-enrichment genes with reduced expression included *IL2RA, ICOS, CXCL13* and *FAS* (**Supplementary Figure 5B**). In addition, CTSG^+^ mast cell abundance was negatively correlated with tumor-infiltrating CD8^+^ T cells and dendritic cells, while positively correlated with B cells, monocytes and neutrophils (**Figure 3D**). Together, these findings support that increased CTSG^+^ mast cell is associated with enhanced tumor metabolic activity alongside impaired chemokine-mediated immune recruitment and reduced CD8^+^ T cell infiltration, potentially contributing to an immune-restricted TME.

We further performed CellChat analysis to predict ligand-receptor interactions between CTSG^+^ mast cells and other cellular components and identified protease-activated receptor (PAR) signaling as a major pathway linking CTSG^+^ mast cells with tumor cells (**Figure 3E**). CTSG predominantly interacted with tumor cells through PAR2 (**Figure 3F**), which belongs to a member of the G protein-coupled receptor family. PAR2 (encoded by *F2RL1*) was mainly expressed in epithelial cells and tumor cells in human CRC tumor tissues (**Figure 3G**). Our previous findings demonstrated that genetic ablation of PAR2 in tumor cells accelerates tumor growth and reduces CXCL9 and CXCL10 production, thereby limiting CD8^+^ T cell recruitment in the TME (22). Notably, CTSG has been reported to cleave PAR2 and subsequently prevent its activation and downstream signaling (23). To further validate the effect of CTSG on PAR2 activation-induced chemokine expression, we treated MC38 cells with purified CTSG and found it significantly suppressed trypsin-induced expression of *Cxcl9*, *Cxcl10* and *Ccl5* (**Figure 3H**). Thus, these findings imply that CTSG could attenuate tumor cell-derived chemokines via PAR2-mediated signaling, thereby limiting CD8^+^ T cell recruitment.

### 4 CTSG reprograms tumor cell metabolism and chemokine signaling

**Figure 4:**
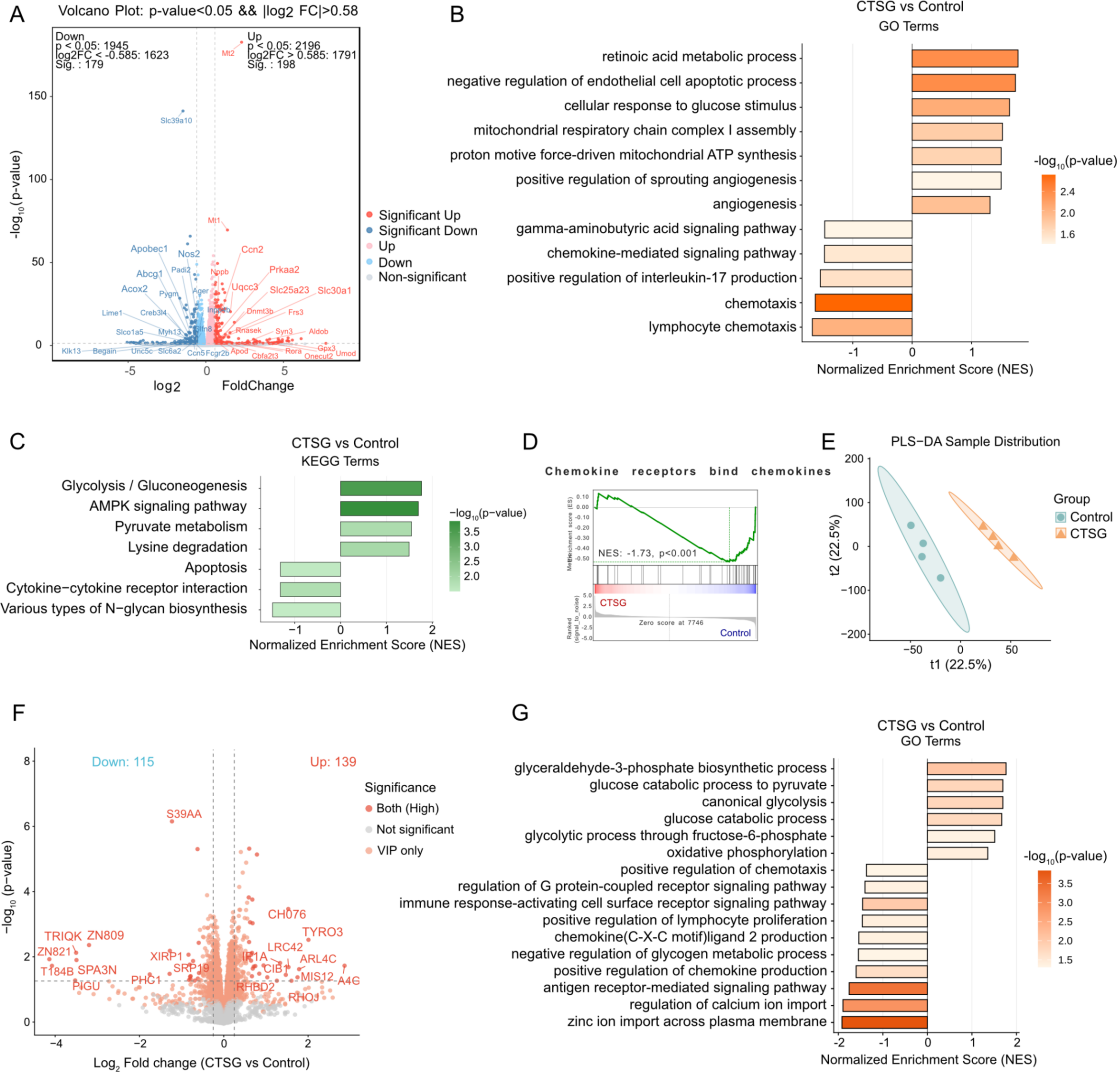
CTSG reprograms tumor cell metabolism and chemokine signaling **(A–D)** Bulk RNA-seq was performed on MC38 cells following treatment with purified CTSG protein or solvent control. **(A)** Volcano plot of differentially expressed genes (DEGs) between the CTSG-treated and control groups. Significantly upregulated and downregulated genes are shown in red and blue, respectively (p < 0.05, |log_2_ (fold change)| > 0.58). GSEA of MC38 cells treated with CTSG vs. control, using GO **(B)** and KEGG **(C)** gene sets. The NES is shown as bar length, and the color scale denotes statistical significance as specified in the figure. **(D)** GSEA plot showing enrichment of chemokine receptors bind chemokines pathway in CTSG-treated group. **(E–G)** Untargeted proteomics was performed on MC38 cells following treatment with CTSG or solvent control. **(E)** Partial least-squares discriminant analysis (PLS-DA) of proteomic profiles. Each point represents an individual sample. The first two components (t1 and t2) are shown, with the percentage of explained variance indicated in parentheses. Ellipses represent the 95% confidence interval for each group. **(F)** Volcano plot integrating log_2_ (Fold change), p-value, and variable importance in projection (VIP) scores. **(G)** GSEA using GO gene sets. The NES is shown as bar length, and the color scale denotes statistical significance as specified in the figure.

To determine the direct effects of CTSG on tumor cells, we treated MC38 cells with purified CTSG and then performed transcriptomic and proteomic analyses. Transcriptomic profiling revealed significant upregulation of multiple metabolism-associated genes involved in mitochondrial respiration, ion transport, and energy sensing, including *Uqcc3*, *Slc25a23*, *Slc30a1*, *Prkaa2*, and *Ccn2*. In contrast, genes associated with inflammatory signaling and metabolic restraint, such as *Nos2*, *Abcg1*, *Apobec1*, and *Acox2* were downregulated (**Figure 4A**), indicating a shift to metabolically active state of tumor cells. GSEA analysis demonstrated that CTSG treatment resulted in enrichment of metabolic pathways, including mitochondrial respiration, ATP synthesis, and cellular responses to glucose (**Figure 4B**). In contrast, pathways involved in chemotaxis and chemokine-mediated signaling, including lymphocyte chemotaxis were markedly suppressed. KEGG analysis revealed enrichment of glycolysis/gluconeogenesis, AMPK signaling, and pyruvate metabolism, as well as suppression of cytokine-cytokine receptor interaction pathways (**Figure 4C**). In addition, pathways related to chemokine signaling were downregulated (**Figure 4D**). Consistent with these pathway-level changes, representative chemotaxis-associated core enrichment genes, including *Cxcl9*, *Ccl2*, and *Ccl9*, were downregulated, whereas energy metabolism-associated genes, including *Ndufb8*, *Ldhb*, and *Aldob*, were upregulated. (**Supplementary Figure 6A**).

Proteomic analysis demonstrated a clear separation between CTSG-treated and control groups, indicating a proteomic shift induced by CTSG (**Figure 4E**). Differential protein analysis identified upregulated proteins, including TYRO3, CH076 and IF1A, were associated with metabolic regulation and cellular stress responses, whereas downregulated proteins, such as SPAN3, ZN809 and TRIQK, were enriched in cellular communication processes (**Figure 4F**). GSEA of differentially expressed proteins revealed enrichment of pathways related to glycolysis, glucose catabolism, and oxidative phosphorylation (**Figure 4G**). In contrast, pathways associated with chemokine production, lymphocyte proliferation, antigen receptor-mediated signaling, and G protein-coupled receptor signaling were suppressed (**Figure 4G**). These were in line with the transcriptomic data and indicated CTSG can enhance metabolic activity and attenuate chemokine signaling in tumor cells.

To assess gene-level concordance between the transcriptomic and proteomic data, we matched 7,526 transcript-protein pairs. RNA and protein fold changes showed a weak positive global correlation (Spearman’s ρ = 0.103, *P* = 2.94 × 10^-^¹^9^), indicating pathway-level convergence but limited gene-level concordance (**Supplementary Figure 6B**). Integrative analysis showed coordinated downregulation of chemotaxis/chemokine-associated proteins (e.g., CMKLR1, POSTN) and upregulation of metabolic-associated proteins (e.g., NDUFAF3, COX6A1) (**Supplementary Figure 6B**).

### 5 CTSG inhibition enhances anti-PD-1 efficacy and restores CD8^+^ T cell effector function

**Figure 5:**
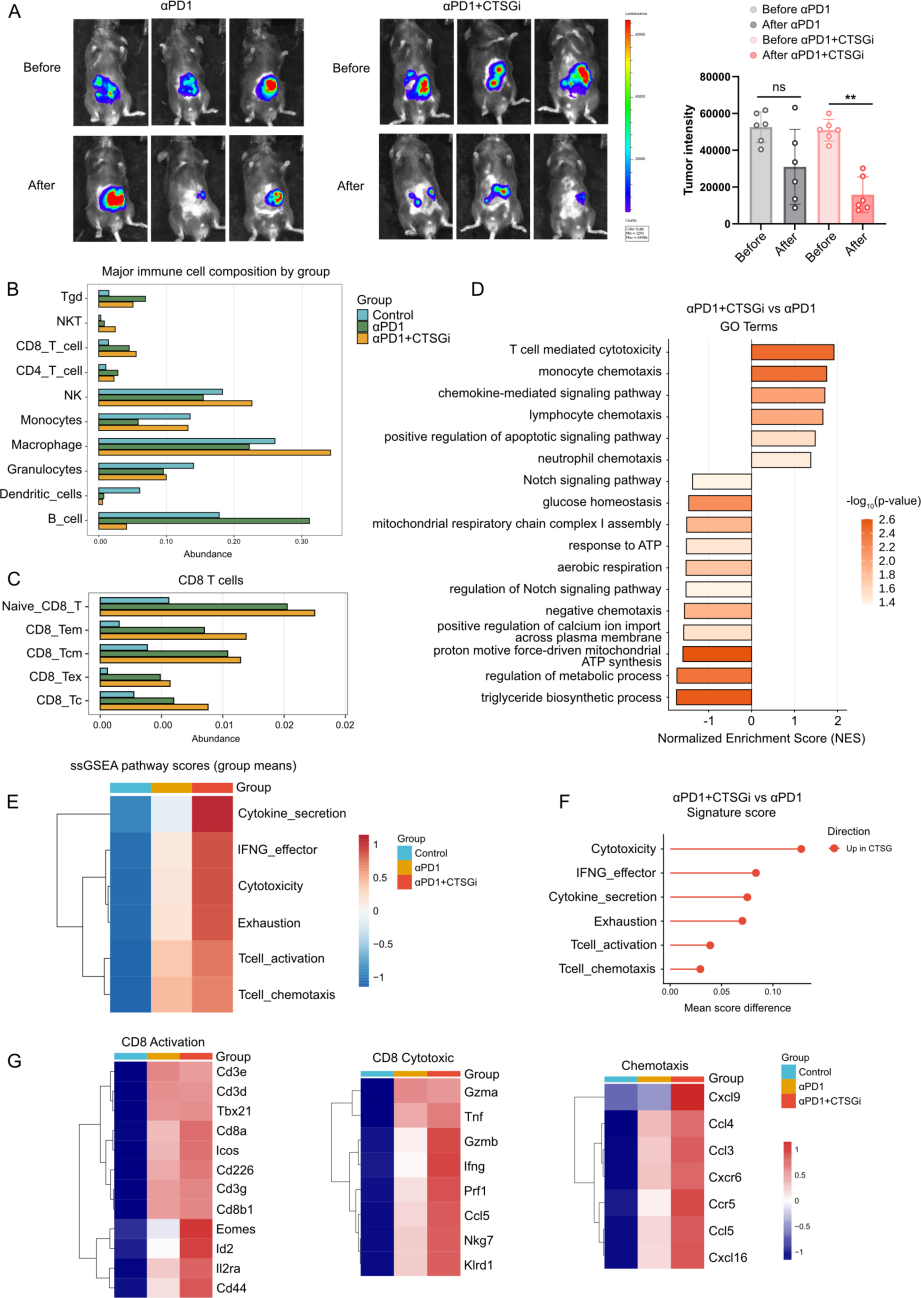
CTSG inhibition enhances anti-PD-1 efficacy and restores CD8^+^ T cell effector function **(A)** Orthotopic CRC model was established by implantation of MC38-Luc cells into the mesenteric side of the cecum of C57BL/6 mice. Mice were treated with anti-PD-1 monotherapy (αPD1) or anti-PD-1 plus CTSG inhibitor (αPD1+CTSGi). Representative images show tumor size and total photon flux within the tumor region. Each dot represents one mouse. Bars indicate the mean ± SD. Statistical significance was assessed using two-sided unpaired *t* test. ** *P* < 0.01, ns, not significant. **(B, C)** Immune cell infiltration was estimated using ImmuCellAI-mouse based on bulk RNA-seq data. Relative infiltration scores of major immune cell populations **(B)** and CD8^+^ T cell subsets **(C)** in each group. Bars represent the estimated infiltration scores for each immune cell type. **(D)** GSEA using GO gene sets between αPD1 and αPD1+CTSGi groups. The NES is shown as bar length, and the color scale denotes statistical significance as specified in the figure. **(E)** ssGSEA pathway scores of tumors from each group. Heatmap displays group mean enrichment scores for indicated pathways. Colors represent row-scaled enrichment scores. **(F)** Comparison of ssGSEA signature scores between αPD1 and αPD1+CTSGi groups. Dot plot showing the difference in mean pathway scores for indicated immune-related signatures. **(G)** Heatmap of CD8^+^ T cell activation and cytotoxic related gene and chemotaxis related gene expression in each group. Values represent group mean expression levels, scaled by row.

We next determined the therapeutic effect of targeting CTSG by using murine orthotopic CRC model. Strikingly, the combination of CTSG inhibitor and anti-PD-1 markedly reduced tumor burden when compared with anti-PD-1 monotherapy, indicating a synergistic antitumor effect (**Figure 5A**). By using ImmuCellAI-mouse tool, we elucidated the tumor-infiltrating immune cells based on bulk RNA-seq data and found that both anti-PD-1 monotherapy and CTSG inhibition plus anti-PD-1 amplified CD8^+^ T cell infiltration, with a more pronounced increase in the combined treatment group (**Figure 5B**). Moreover, effector memory (CD8_Tem) and cytotoxic T cell (CD8_Tc) populations were significantly increased (**Figure 5C**). Functional enrichment analysis revealed that the combined treatment resulted in enrichment of T cell-mediated cytotoxicity, monocyte and lymphocyte chemotaxis, and chemokine-mediated signaling (**Figure 5D**). In contrast, several metabolic pathways including aerobic respiration and ATP synthesis were downregulated. ssGSEA-based pathway scoring also revealed upregulation of CD8^+^ T cell-related functional programs in response to combined therapy, especially the cytotoxicity and IFN-γ response pathways (**Figures 5E, F**). Moreover, the heatmap revealed that key genes associated with CD8^+^ T cell activation (*Cd8a, Cd44, Icos*), cytotoxicity (*Gzmb, Ifng, Prf1*), and immune recruitment (*Cxcl9, Ccl4, Ccl5, Cxcr6*) were significantly upregulated in combined treatment group compared with anti-PD-1 alone (**Figure 5G**). Collectively, these findings demonstrate that CTSG inhibition not only enhances CD8^+^ T cell infiltration and function but also creates a permissive immune environment that improves anti-PD-1 efficacy, supporting the rationale for combined approaches in treating CRC.

## Discussion

In this study, we identify a crucial link between intratumoral mast cells and ICI efficacy in CRC, revealing a distinct CTSG^+^ subset that correlates with poor outcomes. Mast cell-derived CTSG is a key regulator of the TME in CRC. CTSG blockade improves the recruitment and function of CD8^+^ T cell and sensitizes tumors to anti-PD-1 therapy, supporting CTSG as a potential therapeutic target in CRC.

The cellular composition in the TME influences tumor progression and the response to immunotherapy. Patients with “cold” tumors, characterized by low immune cell infiltration or an immunosuppressive state, have a poor response to ICI therapy, whereas those with immune-active “hot” tumors usually respond effectively (5). Therefore, converting “cold” tumors into “hot” tumors is a promising strategy to improve the efficacy of ICIs. Previous approaches to address this include enhancing the immunogenicity of tumor cells, augmenting the function of antigen-presenting cells and effector T cells, eliminating or reprogramming immunosuppressive cells, and disrupting physical barriers to increase T cell infiltration (24). However, these strategies have little effect on reversing ICI resistance in CRC, and it is imperative to explore new therapeutic targets to remodel the TME.

In recent years, the immune regulatory role of mast cells in the TME has received increasing attention. Mast cells are characterized as a “few but efficient” population and possess unique characteristics that distinguish them from other immune cells: (i) as long-lived tissue-resident cells, they do not rely on chemokine recruitment and can exert early, localized, and persistent effects during tumor progression; (ii) their cytoplasm contains abundant pre-stored secretory granules, which can be rapidly released upon external stimulation to reshape the local microenvironment; (iii) through the diverse mediators they release, mast cells can broadly interact with various cells in the microenvironment, exerting pleiotropic regulatory functions. These features make mast cells a promising target for tumor immunotherapy. Stabilizing mast cell degranulation by cromolyn or reducing mast cells by imatinib were reported to inhibit tumor growth and enhance ICI efficacy in solid tumors (20). The role of mast cells in CRC is complex and bidirectional, and their function in the context of immunotherapy has remained unclear. We found that although there was no significant change in the proportion of mast cells in CRC tumor tissues, they exhibited increased transcriptomic complexity. Notably, tumor-infiltrating mast cells were closely associated with the molecular features and immunotherapy response in CRC patients. By using an orthotopic CRC model, we found that mast cell deficiency significantly attenuated tumor growth and increased immune cell infiltration, consisting with previous reported pro-tumoral effects of mast cell in AOM/DSS-induced CRC model (11). Thus, mast cells are not merely passive bystanders, but active regulators of the TME in CRC.

The emerging recognition of mast cells’ multifaceted roles in tumors has led to the identification of a spectrum of mast cell types (21). For instance, IL-4^+^ mast cells promote the formation of a fibrotic TME in triple-negative breast cancer, thereby limiting lymphocyte infiltration and contributing to a “cold” tumor formation and ICI resistance (25). In contrast, a mast cell subset with antigen-presenting capacity in triple-negative breast cancer can activate tumor-reactive T cells, and its abundance is positively associated with ICI efficiency (20). Previous study has identified the presence of CTSG^+^ mast cells in CRC (26), but the specific clinical and functional relevance of this subset remained unclear. In this study, we demonstrated that CTSG^+^ mast cells were significantly enriched in MSS tumors and associated with poor response to immunotherapy in CRC. We also found CD74^+^ mast cell subset, characterized by antigen-presenting capacity, was slightly decreased in CRC patients who do not completely respond to immunotherapy, consisting with the previous studies (20). These results revealed mast cell heterogeneity and their distinct immune regulatory roles in the TME of CRC. Furthermore, increased CTSG^+^ mast cell infiltration was associated with enhanced tumor metabolic activity as well as impaired chemokine-mediated immune recruitment and reduced CD8^+^ T cell presence. These findings align with the notion that metabolic reprogramming is not merely a consequence of tumor growth but a key driver of immune evasion. Metabolic competition in the TME can limit nutrient availability, alter cytokine landscapes, and ultimately suppress antitumor immune responses (27). Our data suggest that CTSG^+^ mast cells may promote the formation of a metabolically active yet immunologically “cold” tumor in CRC, therefore affecting the efficacy of immunotherapy.

Cell-cell communication analysis based on CRC single-cell transcriptomic data indicated that interactions between CTSG^+^ mast cells and tumor cells were predominantly mediated by PAR2, which is broadly expressed by various cell types in colon and plays key roles in regulating barrier integrity, ion transport and glandular secretion (28). PAR2 is activated by serine proteases including trypsin and tryptase, which can cleave the extracellular N-terminus of PAR2 and unmask of a neoepitope termed the tethered ligand to elicit downstream signaling. Our previous studies have revealed an antitumor role of PAR2 in CRC (22, 29). PAR2 deficiency accelerates tumor growth and impairs the responsiveness to anti-PD-1. PAR2 activation enhanced CXCL9 and CXCL10 expression via PI3K-AKT pathway, facilitating CD8^+^ T cell recruitment and activation in tumor. Notably, CTSG can act as a serine protease to cleave the tethered ligand downstream of PAR2, thereby inactivating the receptor and silencing PAR2-dependent signaling (23). Consistently, we showed that CTSG significantly reduced PAR2 activation-induced CXCL9 and CXCL10 expression. These findings suggest that CTSG may modulate TME in CRC via inactivating PAR2 signaling. Our scRNA-seq data showed that CTSG was specifically expressed in mast cells, while its expression was almost undetectable in other cells in CRC, suggesting CTSG may be a therapeutic target. Indeed, by an orthotopic CRC model, we demonstrated that CTSG inhibition significantly enhanced the efficacy of anti-PD-1 therapy. This effect was accompanied by increased infiltration of CD8^+^ T cells, enrichment of cytotoxicity and IFNγ related programs, and induction of T cell chemokines, suggesting that CTSG inhibition can shift the TME toward a more immune-permissive contexture. Our finding also highlights that selective inhibition of mast cell-derived CTSG would provide more precise modulation of immunosuppressive pathways, while preserving the beneficial functions of mast cells.

Several limitations should be acknowledged. First, scRNA-seq analyses relied on original dataset annotations, and the metadata of the adjacent normal tissues did not allow layer-specific comparisons with tumor tissues. Second, intraperitoneal administration of CTSG inhibitor cannot distinguish local TME effects from systemic immune modulation. Third, pharmacological inhibition did not prove that the observed effects were specifically attributable to mast cell-derived CTSG. Addressing these questions will require future studies using local delivery approach and mast cell-specific CTSG perturbation.

## Data Availability

The sequencing data generated and analysed during the current study are available in the GSA database under the accessions CRA041462, CRA041522 and CRA041581. The proteomics data generated in this study have been deposited in the OMIX database under the accession number OMIX017769. The scRNA-seq data were obtained from the Single-cell Colorectal Cancer Atlas (https://crc.icbi.at/) (17) and GEO database (accession number GSE236581) (18).
