# Supplementary material for: CTSG-expressing mast cells confer resistance to immunotherapy in colorectal cancer
Source: Front Oncol. 2026 Jul 1;16:1854253. doi: 10.3389/fonc.2026.1854253 (PMC13368519; doi:10.3389/fonc.2026.1854253)
Supplement: Supplementary file 1 [file DataSheet1.docx]

**Supplementary Figure 1**

**
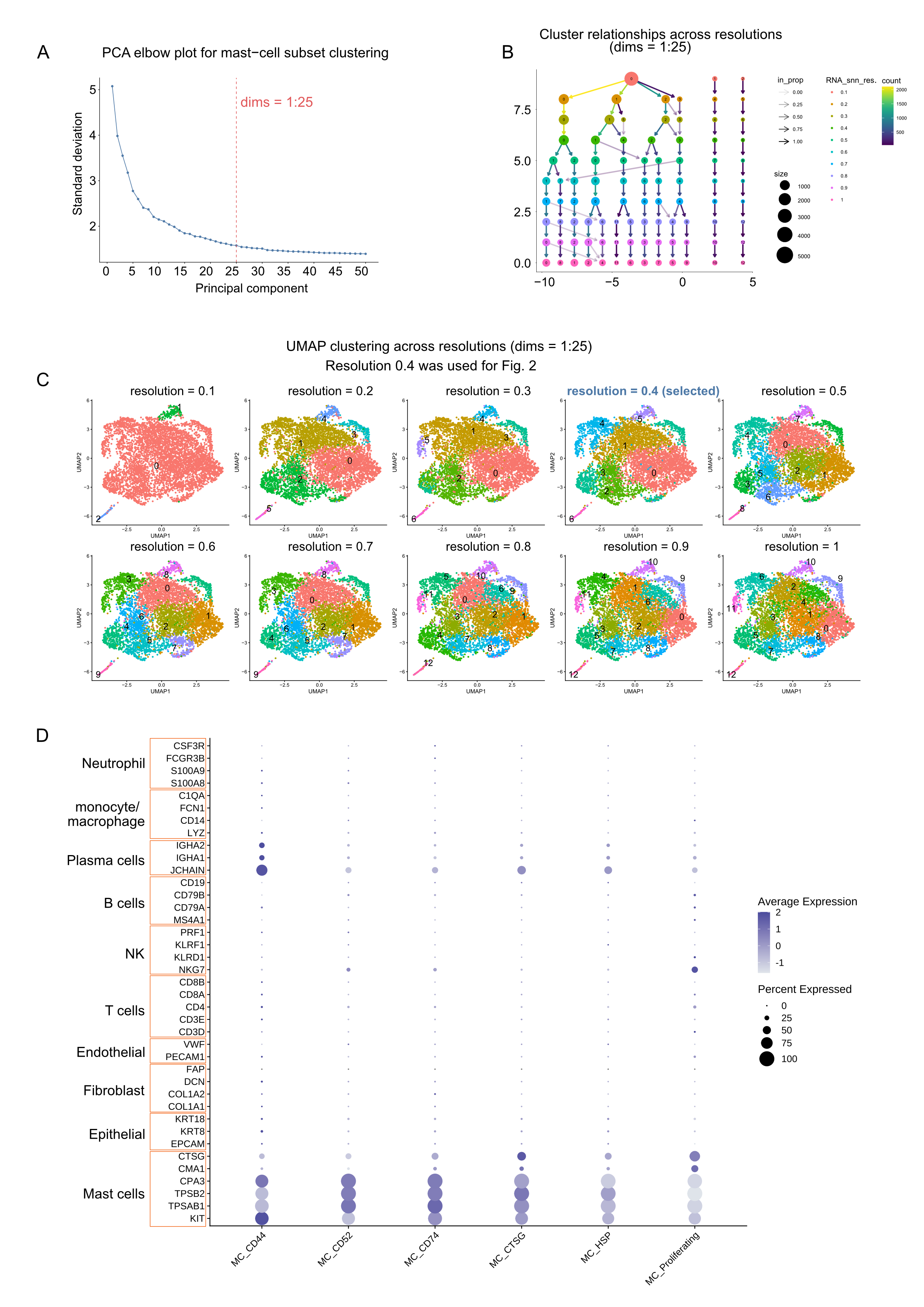
**

**Supplementary Figure 1. Parameter selection, clustering stability and annotation of mast cell subsets in CRC**

**A,** PCA elbow plot for mast cell subclustering. The first 25 principal components were used for downstream analysis. **B,** Clustree visualization of cluster relationships across graph-based clustering resolutions from 0.1 to 1.0 using the first 25 principal components. **C,** UMAP visualizations of mast cell clustering across resolutions from 0.1 to 1.0. A resolution of 0.4 was selected for downstream analyses. **D,** Dot plot assessing potential contamination of mast cells by other major cell lineages.

**Supplementary Figure 2**

**
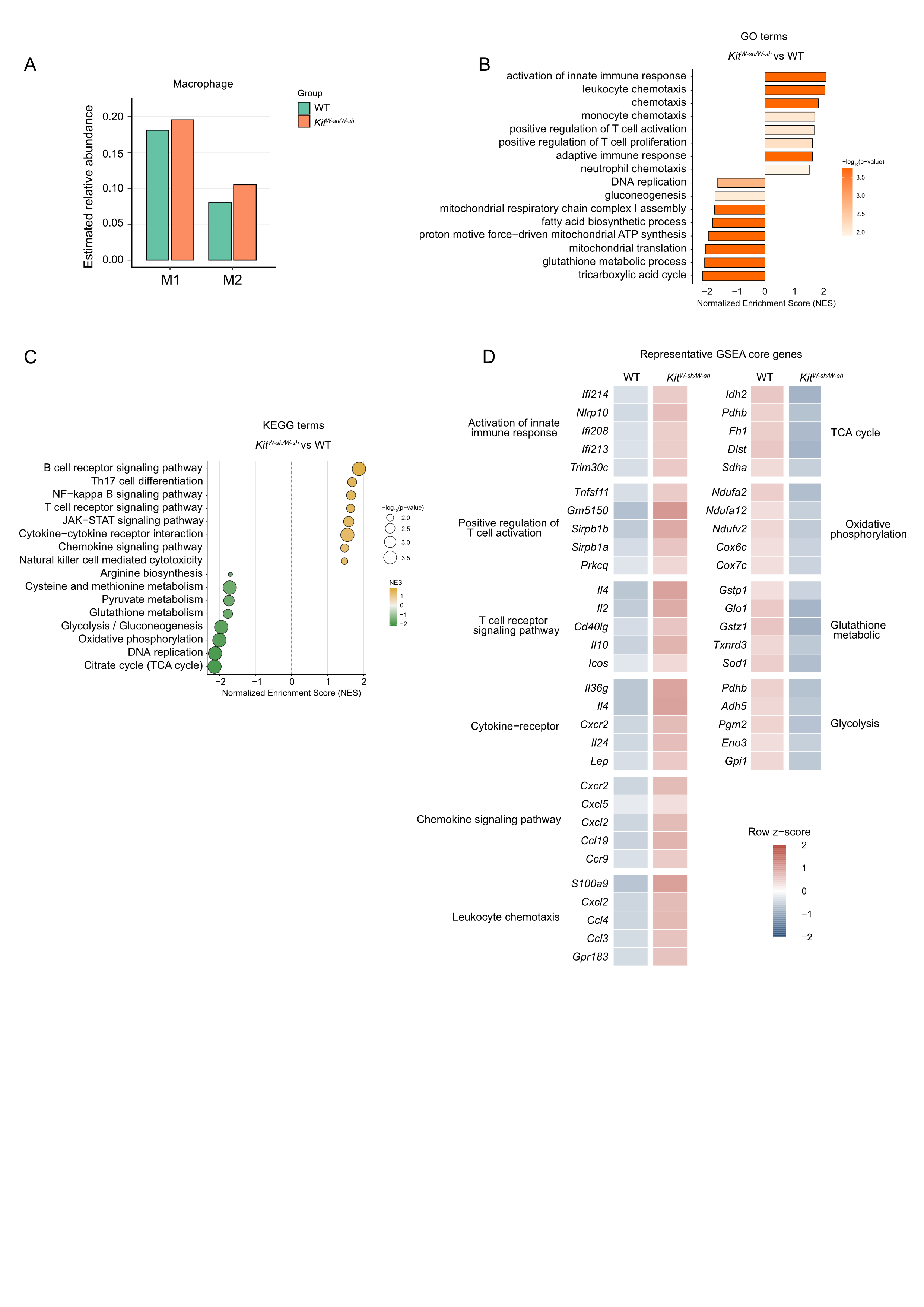
**

**Supplementary Figure 2.** **Mast cell deficiency is associated with altered immune-related and metabolic transcriptional programs in orthotopic CRC tumors**

**A**, ImmuCellAI-mouse-inferred relative abundance estimates of M1- and M2-like macrophages in tumors from WT and *Kit^W-sh/W-sh^* mice. **B**, GO-based GSEA of bulk RNA-seq data from tumor of WT and *Kit^W-sh/W-sh^* mice. **C**, KEGG-based GSEA of bulk RNA-seq data from tumors of WT and *Kit^W-sh/W-sh^* mice. **D**, Heatmap showing representative core-enrichment genes contributing to selected immune-related and metabolic pathways. Values represent row-scaled group-mean expression levels (row *z*-scores) in tumors from WT and *Kit^W-sh/W-sh^* mice.

**Supplementary Figure 3**

**
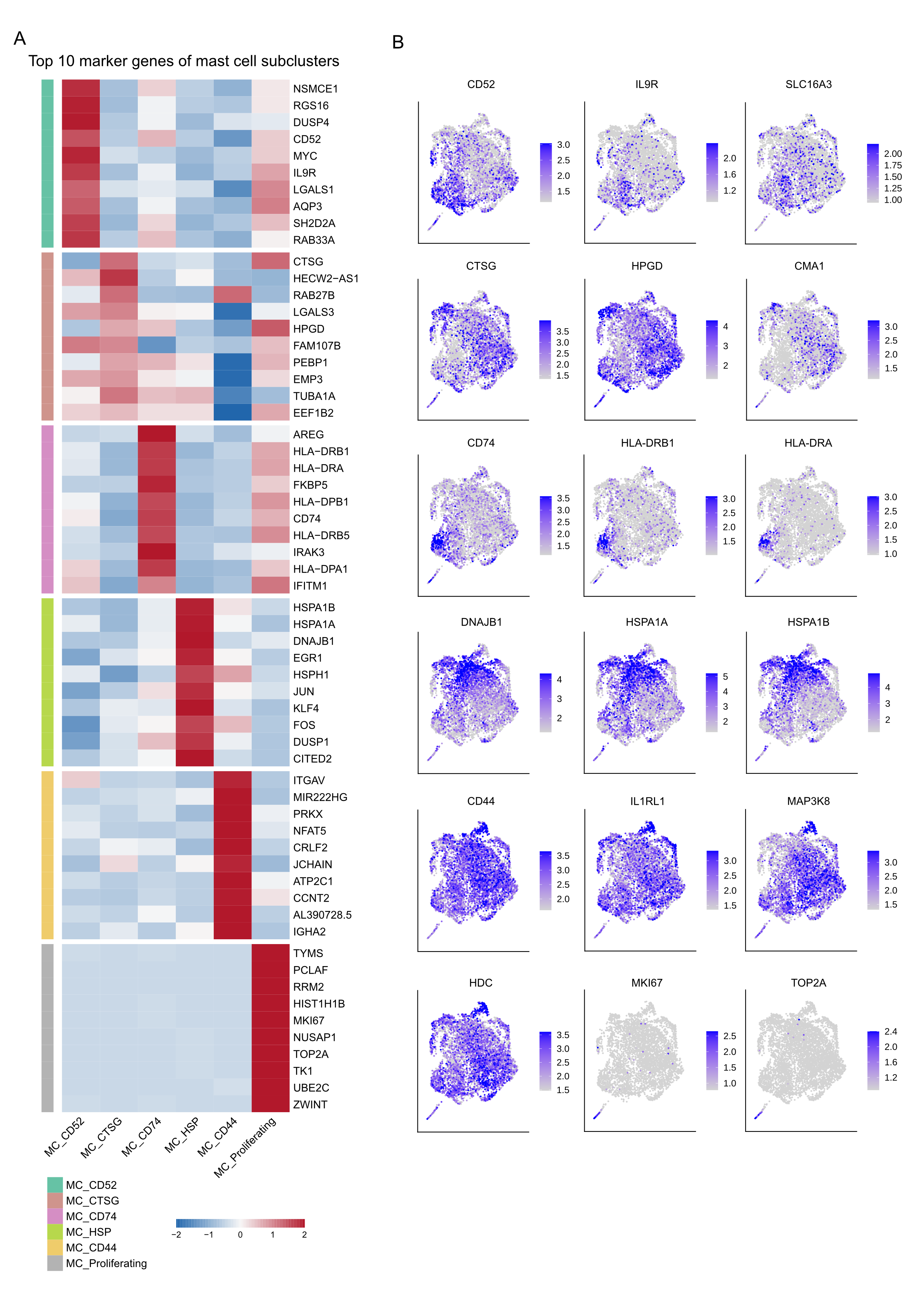
**

**Supplementary Figure 3.** **Marker-gene expression supports the annotation of transcriptionally distinct mast cell states in CRC**

**A**, Heatmap showing the top 10 differentially expressed marker genes for each mast cell subcluster identified by unsupervised clustering of CRC scRNA-seq data. Columns represent the indicated mast cell states, and rows represent marker genes. Values are scaled by row (*z*-scores). **B**, UMAP feature plots showing the expression of representative genes used to support mast cell state annotation. Expression levels are indicated by the color scales shown beside each plot.

**Supplementary Figure 4**

**
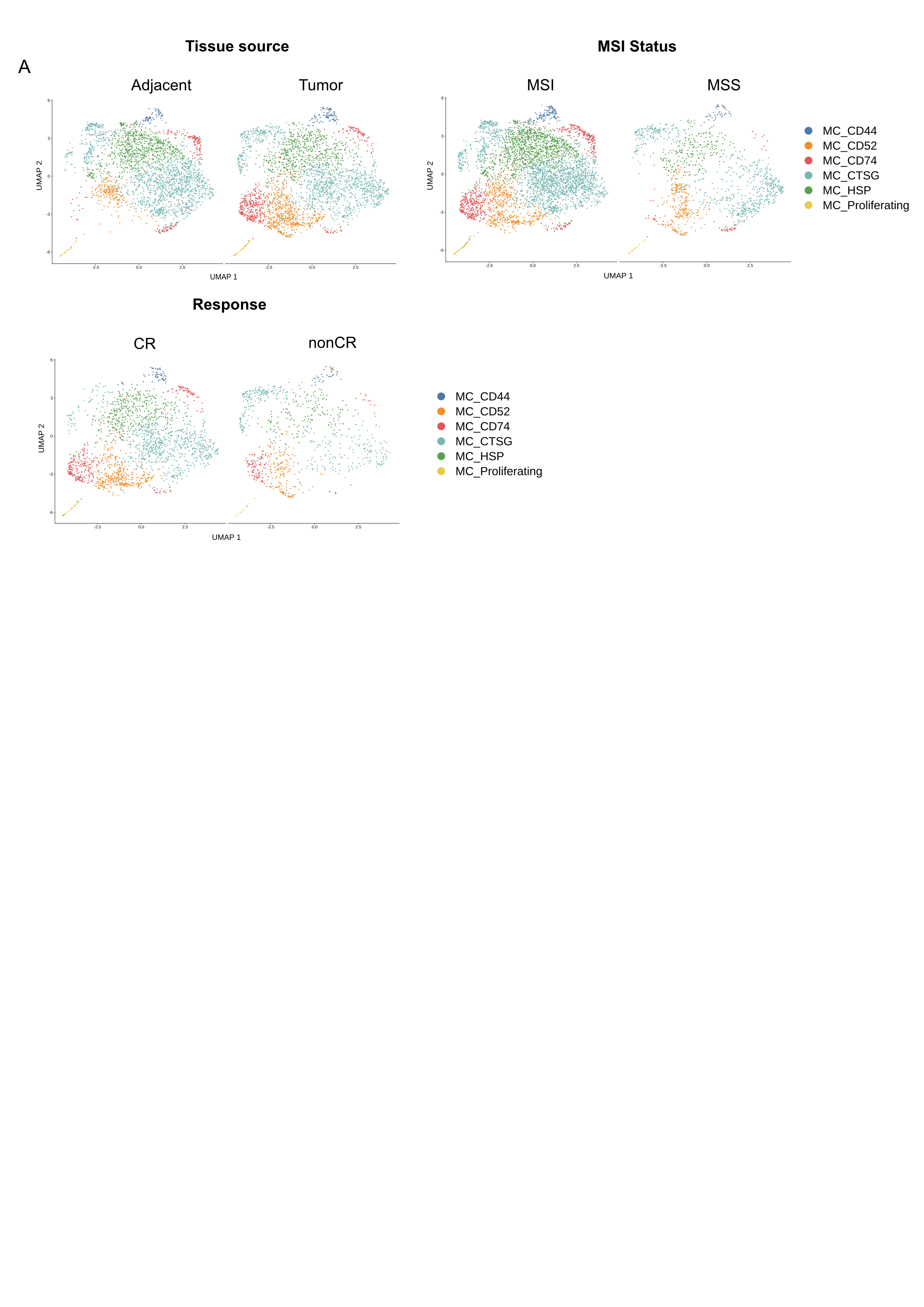
**

**Supplementary Figure 4. Distribution of transcriptionally distinct mast cell states across CRC tissues and clinical groups**

**A**, UMAP visualization of mast cells from CRC scRNA-seq datasets, stratified by tissue source (tumors and adjacent normal tissues), microsatellite-instability status (MSI and MSS), and response to immune checkpoint inhibitor therapy (complete response, CR; and non-complete response, non-CR).

**Supplementary Figure 5**


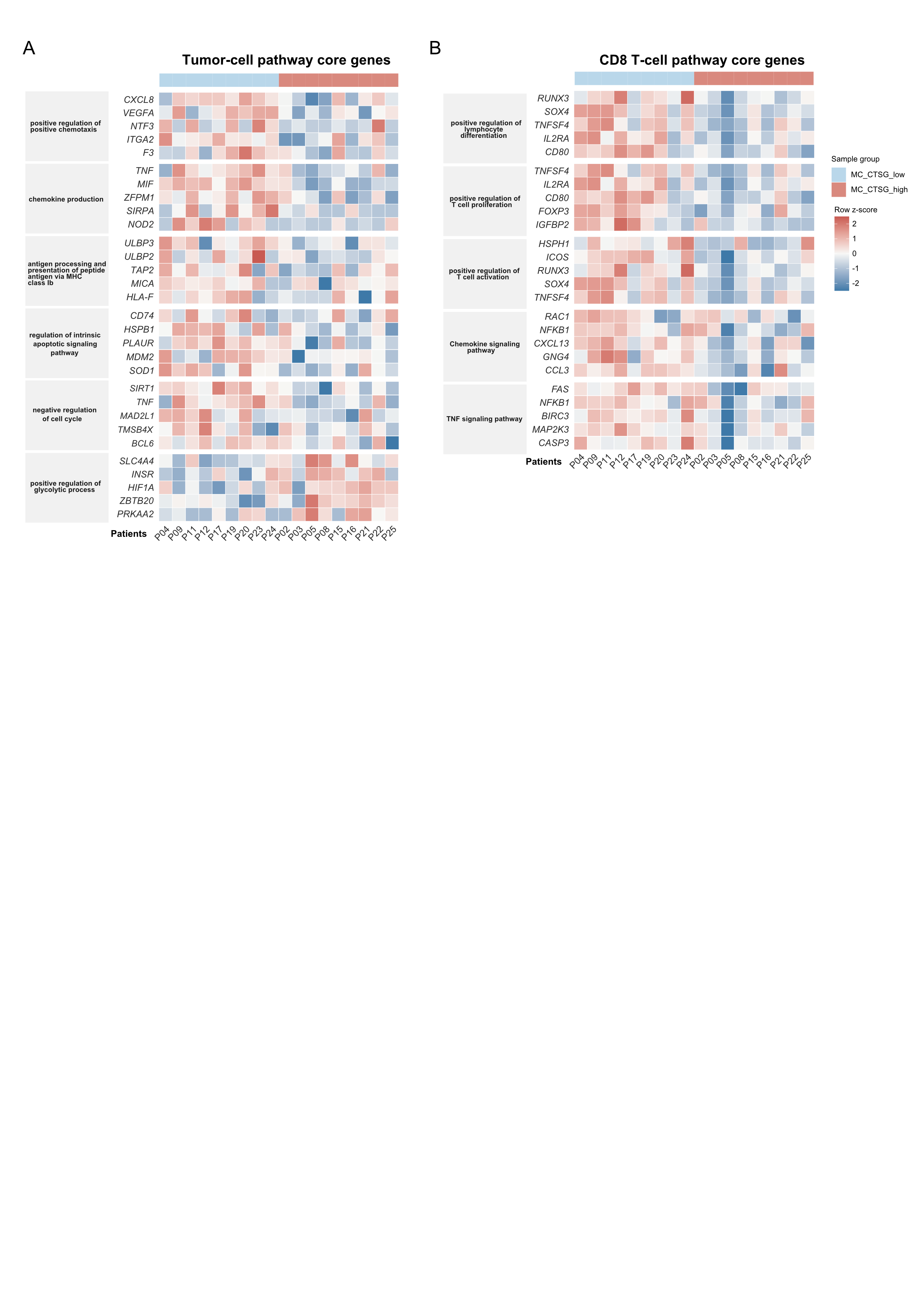


**Supplementary Figure 5. MC_CTSG abundance is associated with altered tumor cell and CD8⁺ T cell transcriptional programs in CRC**

**A**, Heatmap showing representative core-enrichment genes from selected pathways in tumor cells from patients with low or high MC_CTSG abundance. **B**, Heatmap showing representative core-enrichment genes from selected pathways in CD8⁺ T cells from patients with low or high MC_CTSG abundance. Patients were stratified according to the median proportion of MC_CTSG cells among total mast cells. Columns represent individual patients, and values represent row-scaled expression levels (row *z*-scores).

**Supplementary Figure 6**


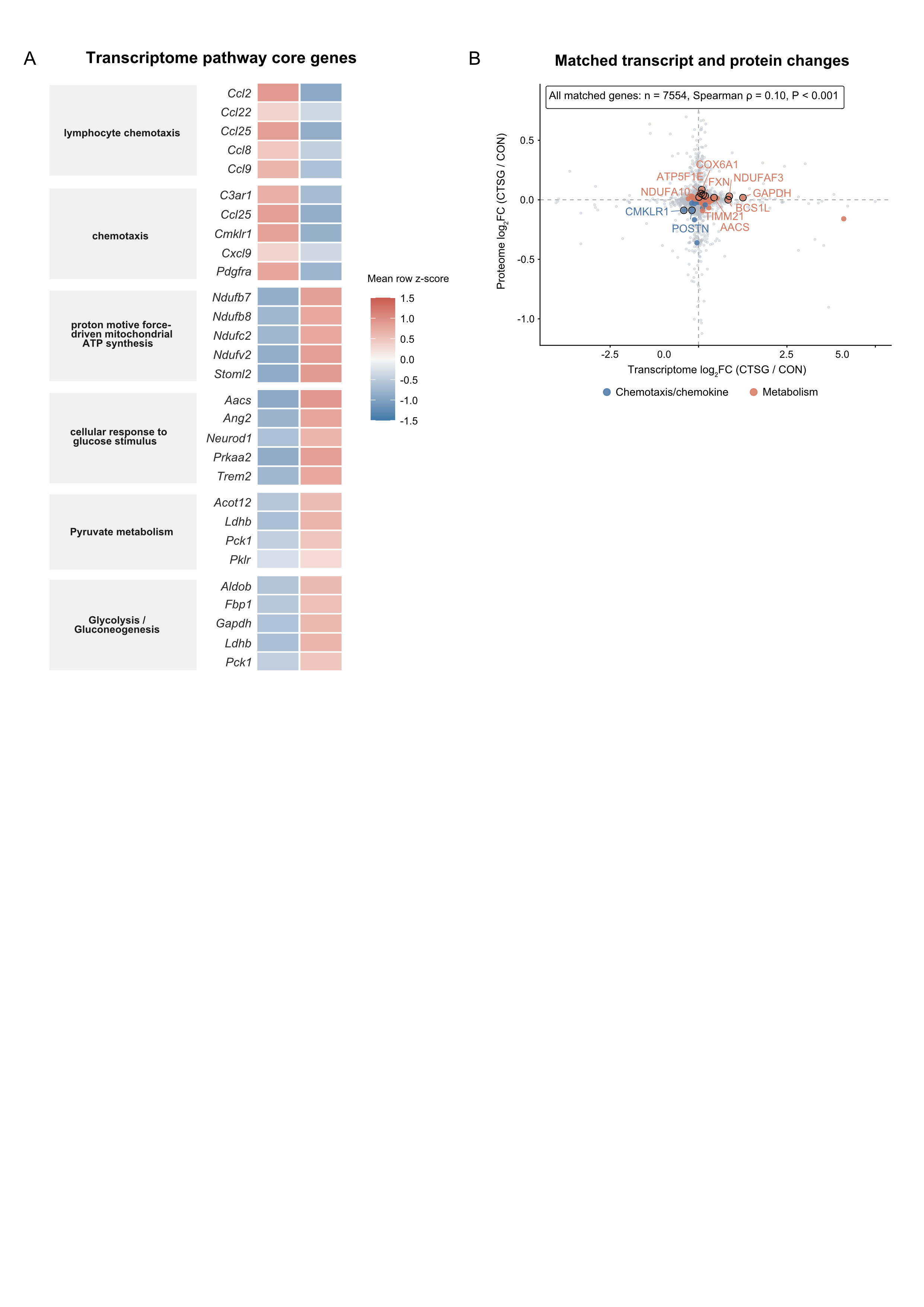


**Supplementary Figure 6. Transcriptomic and proteomic profiling reveals pathway-level convergence but limited gene-level concordance upon CTSG treatment**

**A**, Heatmap showing representative transcriptomic core-enrichment genes from selected pathways in control and CTSG-treated MC38 cells. **B**, Scatter plot comparing transcriptomic and proteomic log₂ fold changes following CTSG treatment. Each point represents a matched transcript–protein pair. Grey points indicate all matched genes, whereas colored points highlight representative chemotaxis/chemokine-related and metabolism-related items.
